# Elimination rates of electrolytes, vitamins and trace elements during continuous renal replacement therapy with citrate CVVHD: influence of treatment dose

**DOI:** 10.1186/cc13584

**Published:** 2014-03-17

**Authors:** P Von Freyberg, W Stahl, H Reinelt, K Träger

**Affiliations:** 1 Klinikum Heidenheim, Germany; 2 University Hospital Ulm, Germany

## Introduction

During continuous renal replacement therapy (CRRT), relevant losses of nutritional substrates, vitamins and trace elements due to diffusive transport and elimination via the filter may occur. We investigated the amount of these losses with regard to treatment dose during a 72-hour treatment period.

## Methods

Forty-one patients with CRRT were investigated. Patients were treated with a citrate CVVHD with different standard doses resulting in a treatment dose range from 14 to 53 (median 27) ml/ kg/hour. Losses were calculated from substrate concentrations in the dialysate × (dialysate + ultrafiltration) flow. During a 72-hour treatment period, each 24 hours were determined separately. Regression analysis was performed for the respective per-day losses related to treatment doses.

## Results

Regression analyses of ER for Ca^2+^, Mg^2+^, PO_4_^2−^, zinc, folic acid and vitamin B12 are shown in Table 1 as the median and interquartile range.

**Table 1:** ER elimination rates

Parameter	Average daily ER	Day 1 *R*^2^	Day 2 *R*^2^	Day 3 *R*^2^
Ca^2+ ^(mmol/day)	97 (83 to 107)	0.2125	0.2448	0.2315
Mg^2+ ^(mmol/day)	6 (3 to 9)	0.0247	0.0708	0.0733
PO_4_^2− ^(mmol/day)	53 (40 to 69)	0.003	0.0556	0.0012
Zinc (μmol/day)	46 (27 to 73)	0.005	0.0527	0.0502
Folic acid (nmol/day)	268 (180 to 388)	0.0935	0.0031	0.0353
Vitamin B12 (pmol/day)	2,060 (1,690 to 2,558)	0.0009	0.0092	0.0152

## Conclusion

Only Ca^2+ ^showed a correlation of ER and treatment dose. Mg^2+^, PO_4_^2− ^zinc, folic acid and vitamin B12 were eliminated without a correlation to treatment dose.

